# A Role for Pre-mRNA-PROCESSING PROTEIN 40C in the Control of Growth, Development, and Stress Tolerance in *Arabidopsis thaliana*

**DOI:** 10.3389/fpls.2019.01019

**Published:** 2019-08-13

**Authors:** Carlos Esteban Hernando, Mariano García Hourquet, María José de Leone, Daniel Careno, Javier Iserte, Santiago Mora Garcia, Marcelo Javier Yanovsky

**Affiliations:** Comparative Genomics of Plant Development Laboratory, Instituto de Investigaciones Bioquímicas de Buenos Aires–Consejo Nacional de Investigaciones Científicas y Técnicas de Argentina, Fundación Instituto Leloir, Buenos Aires, Argentina

**Keywords:** pre-mRNA processing protein 40, splicing, transcription, *Arabidopsis*, biotic stress, abiotic stress

## Abstract

Because of their sessile nature, plants have adopted varied strategies for growing and reproducing in an ever-changing environment. Control of mRNA levels and pre-mRNA alternative splicing are key regulatory layers that contribute to adjust and synchronize plant growth and development with environmental changes. Transcription and alternative splicing are thought to be tightly linked and coordinated, at least in part, through a network of transcriptional and splicing regulatory factors that interact with the carboxyl-terminal domain (CTD) of the largest subunit of RNA polymerase II. One of the proteins that has been shown to play such a role in yeast and mammals is pre-mRNA-PROCESSING PROTEIN 40 (PRP40, also known as CA150, or TCERG1). In plants, members of the PRP40 family have been identified and shown to interact with the CTD of RNA Pol II, but their biological functions remain unknown. Here, we studied the role of AtPRP40C, in *Arabidopsis thaliana* growth, development and stress tolerance, as well as its impact on the global regulation of gene expression programs. We found that the *prp40c* knockout mutants display a late-flowering phenotype under long day conditions, associated with minor alterations in red light signaling. An RNA-seq based transcriptome analysis revealed differentially expressed genes related to biotic stress responses and also differentially expressed as well as differentially spliced genes associated with abiotic stress responses. Indeed, the characterization of stress responses in prp40c mutants revealed an increased sensitivity to salt stress and an enhanced tolerance to *Pseudomonas syringae* pv. *maculicola* (*Psm*) infections. This constitutes the most thorough analysis of the transcriptome of a prp40 mutant in any organism, as well as the first characterization of the molecular and physiological roles of a member of the PRP40 protein family in plants. Our results suggest that PRP40C is an important factor linking the regulation of gene expression programs to the modulation of plant growth, development, and stress responses.

## Introduction

Plants are permanently subjected to environmental changes that adversely affect their growth and development either in natural or agricultural settings. Accurate control of gene expression and precursor mRNAs (pre-mRNAs) processing is essential for the adaptation to rapid changes in the environment. Synthesis of mRNA by the RNA polymerase II (RNA Pol II) is coordinated with subsequent RNA processing events such as 5’ capping, splicing, cleavage, and polyadenylation (Darnell, 2013). The largest subunit of the RNA Pol II couples transcription and pre-mRNA processing *via* its C-terminal domain (CTD). The CTD comprises tandem Tyr1–Ser2–Pro3–Thr4–Ser5–Pro6–Ser7 heptapeptide repeats that are highly conserved in eukaryotes (Allison et al., 1988; Nawrath et al., 1990) and interacts with capping, splicing, and polyadenylation factors, acting as a scaffolding platform for mRNA processing factors (Greenleaf, 1993; McCracken et al., 1997; Misteli and Spector, 1999; Buratowski, 2003; Phatnani and Greenleaf, 2006; Muñoz et al., 2010; Braunschweig et al., 2013).

Pre-mRNA splicing occurs in two sequential trans-esterification steps catalyzed by the spliceosome, a large dynamic ribonucleoprotein complex present in the nucleus. The major spliceosome consists of five small nuclear ribonucleoproteins (snRNPs) named U1, U2, U4, U5, and U6, along with ∼200 accessory proteins (Wahl et al., 2009). The splicing reaction begins with the initial recognition of the 5’ and 3’ splice sites (5’SS and 3’SS, respectively) at the exon–intron boundaries, the branch point sequence (BPS), and the polypyrimidine tract (Py) (Wahl et al., 2009; Meyer et al., 2015); the completion of the splicing process involves the removal of an intron and the ligation of the resulting exons.

Splicing can be classified as constitutive splicing (CS) or alternative splicing (AS), depending on splice site choice and usage. In constitutive splicing, canonical splice sites are always used for a given transcript. In contrast, alternative splicing involves variable splice site choice and/or usage, giving place to multiple mRNAs variants from a single gene. Splice sites can be strong or weak depending on the degree in which their sequences diverge from consensus sequences, and this determines their affinities for the spliceosomal machinery. In general, strong splice sites lead to constitutive splicing through full usage of the site. On the other side, weak splice sites are usually associated with alternative splicing, and the frequency of usage of the alternative splice sites varies depending on cellular context and environmental conditions (Kornblihtt et al., 2013). Alternative splicing events comprise intron retention (IR), exon skipping (ES), and alternative 5’ donor and 3’ acceptor splice site selection (Alt 5’SS and Alt 3’SS, respectively).

In plants of the model species *Arabidopsis thaliana*, current estimates indicate that ∼61% of the intron-containing genes undergo alternative splicing (Marquez et al., 2012), which proved to be essential for proper growth and development, as well as for optimal responses to environmental changes (Reddy et al., 2013; Staiger and Brown, 2013; Ding et al., 2014; Kwon et al., 2014; Shikata et al., 2014; Filichkin et al., 2015; Deng and Cao, 2017). One of the earliest events in spliceosome assembly is the formation of an RNA duplex between the 5’ SS and the 5’ end of the U1 snRNP, an event involving the activity of multiple proteins that stabilize the complex. PRE-mRNA-PROCESSING PROTEIN 40 (PRP40) was first discovered in yeast and proved to be essential in the early steps of spliceosome complex formation (Kao and Siliciano, 1996). PRP40 harbors two WW domains in the amino terminus and four FF domain repeats in the carboxyl terminus, which have been well characterized as mediators of protein–protein interactions (Bedford and Leder, 1999; Macias et al., 2002). In yeast, as well as in mammals, PRP40 helps to define the bridging interaction that links both ends of the intron, by interacting simultaneously with the BRANCHPOINT BINDING PROTEIN (BBP) and the U1 snRNP (Abovich and Rosbash, 1997). *Saccharomyces cerevisiae* PRP40 (ScPRP40) and its three human homologues, HsPRPF40A, HsPRPF40B, and HsTCERG1/CA150, have been extensively studied in the last two decades. While HsPRPF40A and HsPRPF40B were characterized as essential components of the early spliceosome assembly process (Lin et al., 2004; Wahl et al., 2009; Becerra et al., 2015), HsTCERG1/CA150 was initially discovered as a transcriptional modulator and later linked to pre-mRNA splicing (Sune et al., 1997; Pearson et al., 2008; Munoz-Cobo et al., 2017). In fact, HsTCERG1/CA150 has been found associated with elongation factors and is present in a complex with the RNA polymerase II *via* the FF domains (Sune et al., 1997; Carty et al., 2000; Sanchez-Alvarez et al., 2006). HsTCERG1/CA150 has also been linked to RNA splicing through its WW domain 2 (WW2), which interacts with the splicing factors SF1, U2AF, and components of the SF3 complex (Goldstrohm et al., 2001; Lin et al., 2004). Furthermore, HsTCERG1/CA150 has been identified in highly purified spliceosomes in multiple studies (Makarov et al., 2002; Rappsilber et al., 2002; Deckert et al., 2006). As previously mentioned, the processes of transcription and pre-mRNA processing are coordinated by the CTD of the RNA pol II. The modular structure of HsTCERG1/CA150, containing the splicing-factor associating WW domains present in the N-terminus and the CTD-associating FF repeats in the C terminus, confers the ideal structure for a protein that couples transcription and splicing. In accordance with this model, both halves of HsTCERG1/CA150 have been shown to be essential for the assembly of higher-order transcription-splicing complexes (Sanchez-Alvarez et al., 2006).

Plant homologues of ScPRP40 were first identified in 2009 by Kang et. al by means of a bioinformatic screening of proteins that interact with the RNA Pol II CTD in *Arabidopsis*. Three proteins were thus identified: AtPRP40A, AtPRP40B, and AtPRP40C (Kang et al., 2009), which interacted with de RNA Pol II CTD (both phosphorylated and nonphosphorylated); at least for AtPRP40B, the authors showed that the WW domains at the amino terminus mediated this interaction (Kang et al., 2009). So far, a characterization of the biological and molecular roles of plant PRP40s is missing. Here, we decided to focus on the characterization of AtPRP40C using a reverse genetics approach and performed a physiological and molecular analysis of the function of this protein in the control of growth, development and stress tolerance, as well as in the regulation of gene expression. We found that AtPRP40C is involved in the regulation of flowering time and photomorphogenic responses. In addition, an RNA-seq analysis revealed that AtPRP40C is associated with the proper control of expression and splicing of abiotic and biotic stress-related transcripts, and, indeed, physiological assays showed that *prp40c* mutants display altered tolerance to salt stress and *Pseudomonas syringae* pv. *maculicola* (*Psm*) infections. Our work is the first physiological and molecular characterization of a member of the PRP40 protein family in plants, which reveals an important role for PRP40C linking the regulation of gene expression and pre-mRNA splicing, and modulating plant growth, development, and stress responses.

## Materials and Methods

### Plant Material

All of the *Arabidopsis* lines used in this study were on the Columbia (Col-0) ecotype background, and the *prp40c-1* (SALK_148319) and *prp40c-2* (SALK-205357) mutants were obtained from the Arabidopsis Biological Research Center (ABRC). Genotypic verification of the mutants was performed *via* PCR analysis using primers as detailed in **Supplementary Table S1**. PRP40C expression levels in the mutant alleles, as well as in wild-type plants, were assessed by reverse transcription followed by a semiquantitative PCR (RT-PCR) using Actin 2 as expression control; primers used are described in the **Supplementary Table S1**.

### Growth Conditions

Plants were grown on soil at 22°C under long days (LD; 16-h light/8-h dark cycles; 80 μmol m^−2^ s^−1^ of white light), 12:12 days (LD 12:12; 12-h light/12-h dark cycles; 80 μmol m^−2^ s^−1^ of white light), short days (SD; 8-h light/16-h dark cycles; 140 μmol m^−2^ s^−1^ of white light), or continuous light (LL; 50 μmol m^−2^ s^−1^ of white light), depending on the experiment.

### Phylogenetic Analysis

Evolutionary analysis was conducted using the maximum likelihood method implemented in MEGA X (Kumar et al., 2018). Sequences were retrieved using TBLASTN with different query sequences from Phytozome (phytozome.jgi.doe.gov), Onekp project at China National GeneBank (db.cngb.org/blast/blast/tblastn/), www.fernbase.org, and the *Klebsormidium nitens* genome portal (http://www.plantmorphogenesis.bio.titech.ac.jp/). All sequences were manually inspected to search for annotation errors. Retrieved sequences with long stretches of indeterminate bases were excluded from the analysis. In the case of sequences derived from transcriptomic surveys (namely, Onekp), care was taken so that only species with the longest contigs for all the genes searched were included in the analysis.

### Flowering Time Analysis

For flowering time experiments, the plants were grown on soil at 22°C under standard long days (LD; 16-h light/8-h dark cycles; 80 μmol m^−2^ s^−1^ of white light), 12:12 days (12-h light/12-h dark cycles; 80 μmol m−2 s^−1^ of white light), continuous light (LL; 50 μmol m^−2^ s^−1^ of white light) or short days (SD; 8-h light/16-h dark cycles; 140 μmol m^−2^ s^−1^ of white light) depending on the experiment. Flowering time was estimated by counting the number of rosette leaves at the time of bolting. These experiments were performed in triplicate with *n* = 16 for each genotype. The statistical analysis was done using a two-tailed Student’s *t*-test.

### Hypocotyl Length Characterization

For hypocotyl length measurements, seedlings were grown on 0.8% agar under complete darkness, continuous red light (1 μmol m^−2^ s^−1^), continuous blue light (1 μmol m^−2^ s^−1^), continuous white light (LL), or under cycles of white light in SD or LD (all white light treatments 1 μmol m^−2^ s^−1^). The final length of the hypocotyls was measured 4 days after germination. Light effects on hypocotyl elongation under continuous red and blue light were calculated normalizing hypocotyl length under each light regime to the hypocotyl length of the same genotype under constant dark conditions. For the white light experiments, absolute values of hypocotyl elongation are shown. These experiments were performed in triplicate with *n* = 20 for each genotype. The statistical analysis was done using a two-tailed Student’s *t*-test.

### Circadian Leaf Movement Analysis

For leaf movement analysis, plants were grown under 16-h light/8-h dark cycles until the appearance of the first pair of leaves. This period is referred to as the entrainment period. In order to measure circadian rhythms in leaf movement, plants were transferred to continuous white light (20 μmol m^−2^ s^−1^) at 22°C. The position of the first pair of leaves was recorded every 2 h for 5–6 days using digital cameras, and the leaf angle was determined using ImageJ software (Schneider et al., 2012). Period estimates were calculated with Brass 3.0 software (Biological Rhythms Analysis Software System, available from http://www.amillar.org) and analyzed with fast Fourier transform nonlinear least squares (FFT-NLLS) using Brass 3.0 software. These experiments were performed in triplicate, with *n* = 8 for each genotype. The statistical analysis was done using a two-tailed Student’s *t*-test.

### Growth Conditions and Protocol Used for cDNA Library Preparation and High-Throughput Sequencing

Three biological replicates with seeds of wild-type (Col-0) and *prp40c-1* mutant allele were sown onto Murashige and Skoog medium containing 0.8% agarose, stratified for 4 days in the dark at 4°C and then grown at 22°C in continuous light. Whole plants were harvested after 12 days, and total RNA was extracted with RNeasy Plant Mini Kit (QIAGEN) following the manufacturer’s protocols. To estimate the concentration and quality of samples, NanoDrop 2000c (Thermo Scientific) and the Agilent 2100 Bioanalyzer (Agilent Technologies) with the Agilent RNA 6000 Nano Kit were used, respectively. Libraries were prepared following the TruSeq RNA Sample Preparation Guide (Illumina). Briefly, 3 μg of total RNA was polyA-purified and fragmented, first-strand cDNA synthesized by reverse transcriptase (SuperScript II, Invitrogen) using random hexamers. This was followed by RNA degradation and second-strand cDNA synthesis. End repair process and addition of a single A nucleotide to the 3′ ends allowed ligation of multiple indexing adapters. Then, an enrichment step of 12 cycles of PCR was performed. Library validation included size and purity assessment with the Agilent 2100 Bioanalyzer and the Agilent DNA1000 kit (Agilent Technologies). Samples were pooled to create six multiplexed DNA libraries, which were pair-end sequenced with an Illumina HiSeq 1500 at INDEAR Argentina, providing 100-bp pair-end reads. Three replicates for each genotype were sequenced. Sequencing data have been uploaded to the Gene Expression Omnibus database and hare available under accession number (GSE129932).

### Processing of RNA Sequencing Reads

Sequence reads were mapped to *A. thaliana* TAIR10 (Lamesch et al., 2012) genome using TopHat v2.1.1 (Trapnell et al., 2009) with default parameters, except of maximum intron length set at 5,000. Count tables for different feature levels were obtained from bam files using custom R scripts and considering TAIR10 transcriptome.

### Differential Gene Expression Analysis

Before differential expression analysis, we decided to discard genes with fewer than 10 reads on average per condition. Differential gene expression was estimated using the edgeR package version 3.4.2 (Robinson et al., 2010), and resulting *p* values were adjusted using a false discovery rate (FDR) criterion (Benjamini and Hochberg, 1995). Genes with FDR values <0.05 and absolute log two-fold change > 0.58 were deemed differentially expressed. Overlapping analysis were performed using Venny (Oliveros, 2007).

### Differential Alternative Splicing

For the analysis of alternative splicing, the transcriptome was partitioned into subgenic joint features called “bins,” as proposed on DEXseq (Anders et al., 2012). Because of our special interest in new intron retention events, not only exons but also introns were considered in our analysis. The transcriptome was partitioned into 281,321 bins; 152,631 corresponding exclusively to exonic regions, 120,717 to intronic regions, and 7,973 to DNA regions directly associated with alternatively spliced events. We labeled these three kinds of bins as exon-bin, intron-bin, or AS-bins, respectively. In addition, AS-bins were further classified as defined by Mancini et al. (2019). For our analysis, we discarded bins from monoexonic genes and with mean count values lower than five reads per condition. We used edgeR exact test for the identification of differential use of bins corresponding to AS events or introns and FDR-corrected *p* values. We also computed read densities to have a relationship between the bin and its corresponding gene. Only genes with read densities > 0.05 in all genotypes were used for the analysis. AS events as well as all introns with an absolute log2 fold change (bin read density in the mutant/bin read density in wild type) value >0.58, with FDR values <0.15 were deemed differentially spliced. Overlapping analysis was performed using Venny (Oliveros, 2007).

### Functional Category Enrichment Analysis

Functional categories associated with specific groups of genes were identified using the BioMaps tool from the virtual plant software (http://virtualplant.bio.nyu.edu/cgi-bin/vpweb). This tool allowed us to determine which functional categories were statistically overrepresented in particular lists of genes compared to the entire genome (Katari et al., 2010). We analyzed 14 functional categories of our interest, and for each one, we determined the genes in common with our data sets, finally calculating a representation factor and the probability of finding an overlap simply by chance. The representation factor is the number of overlapping genes divided by the expected number of overlapping genes drawn from two independent groups. A representation factor  > 1 indicates more overlap than expected by chance for two independent groups of genes or events, a representation factor <1 indicates less overlap than expected. The probability of each overlapping was determined using the hypergeometric probability formula.

### Analysis of Splice-Site Sequences

To evaluate possible changes in the splice-site sequences of the most significantly affected splicing events in the *prp40c-1* mutants, we obtained the donor and acceptor splice site sequences of all the intron retention events that were deemed differentially spliced (absolute log2 fold value >0.58, with FDR values <0.15) in *prp40c-1* mutants compared to wild-type plants and compared them to the consensus splice-site sequences of the total 30,142 introns sequenced in our RNA-seq experiment. The frequency of each nucleotide for each position was obtained using custom R scripts and was represented using the R package Seqlogo (Bembom, 2014). The over- or underrepresentation of a particular nucleotide relative to its genome-wide frequency was determined, and a *p* value for the analysis was obtained using the hypergeometric test. The custom R scripts used here are available upon request.

### Salt and Osmotic Stress Assays

For germination under salt stress assays, 40 seeds of wild-type (Col-0), *prp40c-1*, and *prp40c-2* plants, and three replicates of each, were sown on 3-mm filter paper (Whatman) and were imbibed in different concentrations of sodium chloride (NaCl) solution (30, 50, 70, and 90 mM) or distilled water. After sowing, the plates were irradiated with far-red light and stratified overnight. On the next day, the plates were irradiated with white light for 1 h and then transferred to dark conditions for 3 days at 22°C. A seed was considered as germinated when the embryonary root protruded through the seed envelope. These experiments were performed in triplicate. The statistical analysis was done using a two-way ANOVA. For growth and survival assays, seeds were germinated on 1/2 MS agar medium. In the case of the root growth assay, 4-day-old seedlings were transferred to 1/2 MS agar containing 100 mM of NaCl or 200 mM of D-mannitol (isosmotic concentrations) and grown vertically for 5 days. The plants were photographed after this period of growth, and their root length was assessed. These experiments were performed in triplicate with *n* = 30 for each genotype. For the NaCl tolerance assay, 4-day-old seedlings were transferred from the germination medium to 1/2 MS agar containing 0 or 160 mM of NaCl, and the survival rate was determined 30 days after the seedlings were transferred. These experiments were performed in triplicate with *n* = 50 for each genotype. The statistical analysis was done using a two-tailed Student’s *t*-test.

### Evaluation of Cold Tolerance

Seeds were sown onto 1/2 MS agar medium, stratified for 3 days at 4°C, and grown at 22°C under continuous light. Four-day-old seedlings were transferred to 1/2 MS agar plates and grown vertically for 5 days at 22°C (control) or for 10 days at 10°C under continuous light. The plants were photographed after this period of growth, and their root length was assessed. These experiments were performed in triplicate with *n* = 30 for each genotype.

### Pathogen Infection Assay

*Pseudomona syringae* pv. *maculicolia* ES4326 strains were grown at 28°C with King’s B medium (20 g protease peptone, 1.5 g K_2_HPO_4_, 6.09 ml 1 M MgSO_4_, and 10 g glycerol per liter) supplemented with rifampicin (100mg/L) and kanamycin (50mg/L) for selection. Freshly cultured bacteria were collected and resuspended to a final concentration of OD600 = 0.0002 in 10 mM MgSO_4_. The bacterial solution was then pressured infiltrated with a 1-ml needleless syringe into the abaxial side of the 8th to 10th leaves of 5- to 6-week-old plants grown under SD conditions. Bacterial growth assays were performed at 48 h postinfection (hpi). Leaves were surface washed in sterile water before carrying out bacterial counts. A disc was punched from each leaf, and then, the three discs corresponding to each plant were placed in 750 μl of 10 mM MgCl_2_ and crushed to release the bacteria. The resulting solution was serial diluted and spot plated on KB plates containing the corresponding antibiotics. The plates were incubated at 28°C for 48 h before counting of the colonies. Log-transformed bacterial growth was statistically analyzed. All bacterial infection experiments were repeated at least three times. The statistical analysis was done using a two-tailed Student’s *t*-test.

### PCR Alternative Splicing and qPCR Differential Expression Assessment

For alternative splicing and differential expression assessment, 12-day-old plants were grown in Murashige and Skoog 0.8% agar medium under continuous white light at 22°C. Total RNA was extracted using TRIzol reagent (Invitrogen, Carlsbad, CA, USA). One microgram of RNA was treated with RQ1 RNase-Free DNase (Promega, Madison, WI, USA) and subjected to retro-transcription with Super Script II Reverse Transcriptase (SSII RT) (Thermo Fisher Scientific, Waltham, MA, USA) and oligo-dT according to manufacturer’s instructions. For alternative splicing, PCR amplification was performed using 1.5 U of Taq polymerase (Invitrogen). Primers used for amplification are detailed in **Supplementary Table S1**. RT-PCR products were electrophoresed and detected by SYBR Green 2%. For qPCR differential expression assessment, cDNAs were then amplified with FastStart Universal SYBR Green Master (Roche, Basel, Switzerland) using the Mx3000P Real Time PCR System (Agilent Technologies, Santa Clara, CA, USA) cycler. The IPP2 (AT3G02780) transcript was used as a housekeeping gene. Quantitative RT-PCR (qRT-PCR) analysis was conducted using the 2−ΔΔCT method (Schmittgen and Livak, 2008). Primer sequences and conditions are available on **Supplementary Table S1**. Three biological samples were measured for each genotype. The statistical analysis was done using a two-tailed Student’s *t*-test.

## Results

### Phylogenetic Analysis of the Pre-mRNA Processing Protein 40 Family

**Figure 1:**
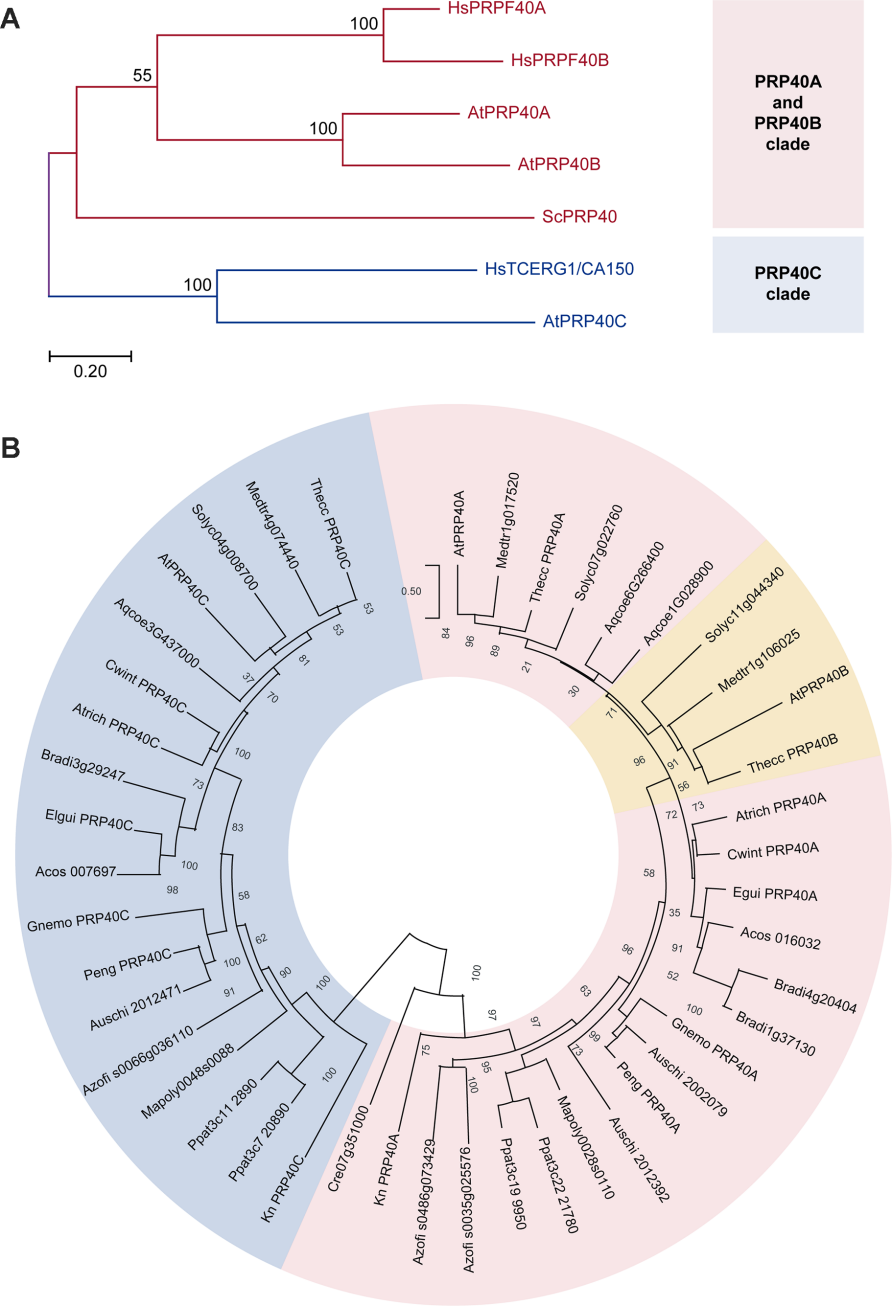
Phylogenetic analysis of the PRP40 family. Pink and blue shades indicate the PRP40A and PRP40C groups, respectively. Numbers indicate the percentage support for each node with 1,000 bootstrap iterations. **(A)** Comparison between the corresponding sequences of yeast (Sc, *Saccharomyces cerevisiae*), humans (Hs, *Homo sapiens*), and Arabidopsis (At, *Arabidopsis thaliana*). **(B)** Phylogenetic relationships of the PRP40 family in the Viridiplantae. The eudicot-specific duplication between A- and B-type sequences is indicated in orange; note that the two sequences from *A. coerulea*, a basal eudicot, do not clearly cluster with either group. Species identification: Medtr, *Medicago truncatula*; Thecc, *Theobroma cacao*, Solyc, *Solanum lycopersicum*; Aqcoe, *Aquilegia coerulea*; Atrich, *Amborella trichopoda*; Cwint, *Canella winterana*; Egui, *Elaeis guineensis*; Acos, *Ananas cosmosus*; Bradi, *Brachipodium distachyon*; Gnemo, *Gnetum montanum*; Auschi, *Austrocedrus chilensis*; Peng, *Picea engelmannii*, Azofi, *Azolla filiculoides*, Ppat, *Physcomitrella patens*; Mapoly, *Marchantia polymorpha*; Kn, *Klebsormidium nitens*; Cr, *Chlamydomonas reinhardtii.*

We initially performed a phylogenetic analysis of the pre-mRNA processing protein 40 family (PRP40) (**Figure 1A**). We found that PRP40s from both human and *Arabidopsis* genomes cluster in two separate clades, one that includes the so-called PRP40A and B types and another that includes HsTCERG1/CA150 and AtPRP40C. In order to assess whether this clustering reflects an ancestral origin, or may be an effect of sequence affinity unrelated to descent, we retrieved and analyzed plant PRP40 sequences sampled from phylogenetically relevant species (**Figure 1B**). We found that members of the PRP40A/B clade are found in all Viridiplantae, both uni- and multicellular. However, the occurrence of A and B forms appear to be restricted to eudicots, since neither monocots nor basal angiosperms show this type of duplication. PRP40C-type sequences are also widespread among plants, usually found as single copy genes. Still, we could not find *bona fide* homologues in Chlorophytes; consequently, PRP40C genes appear to be restricted to streptophytes. Taken together, our analysis shows that 1) type A PRP40s appear to be an ancestral feature of eukaryotic organisms, 2) the occurrence of duplicated forms in this clade (types A and B) is lineage specific, and 3) genes of the HsTCERG1/PRP40C type may also have independent origins; in Viridiplantae, they appear to be an ancient innovation of streptophytes.

Thus, both AtPRP40C and HsTCERG1/CA150 appear to have diverged from the others members of the family in the respective organism. This observation, together with the fact that HsTCERG1/CA150 is a well-characterized protein that links transcription and pre-mRNA processing, suggested that this gene could have evolved to modulate gene expression networks in complex eukaryotic organisms and prompted us to analyze its biological roles and molecular functions in *A. thaliana*.

### PRP40C Mutants Display a Late Flowering Phenotype and Mild Alterations in Other Light Controlled Process

**Figure 2:**
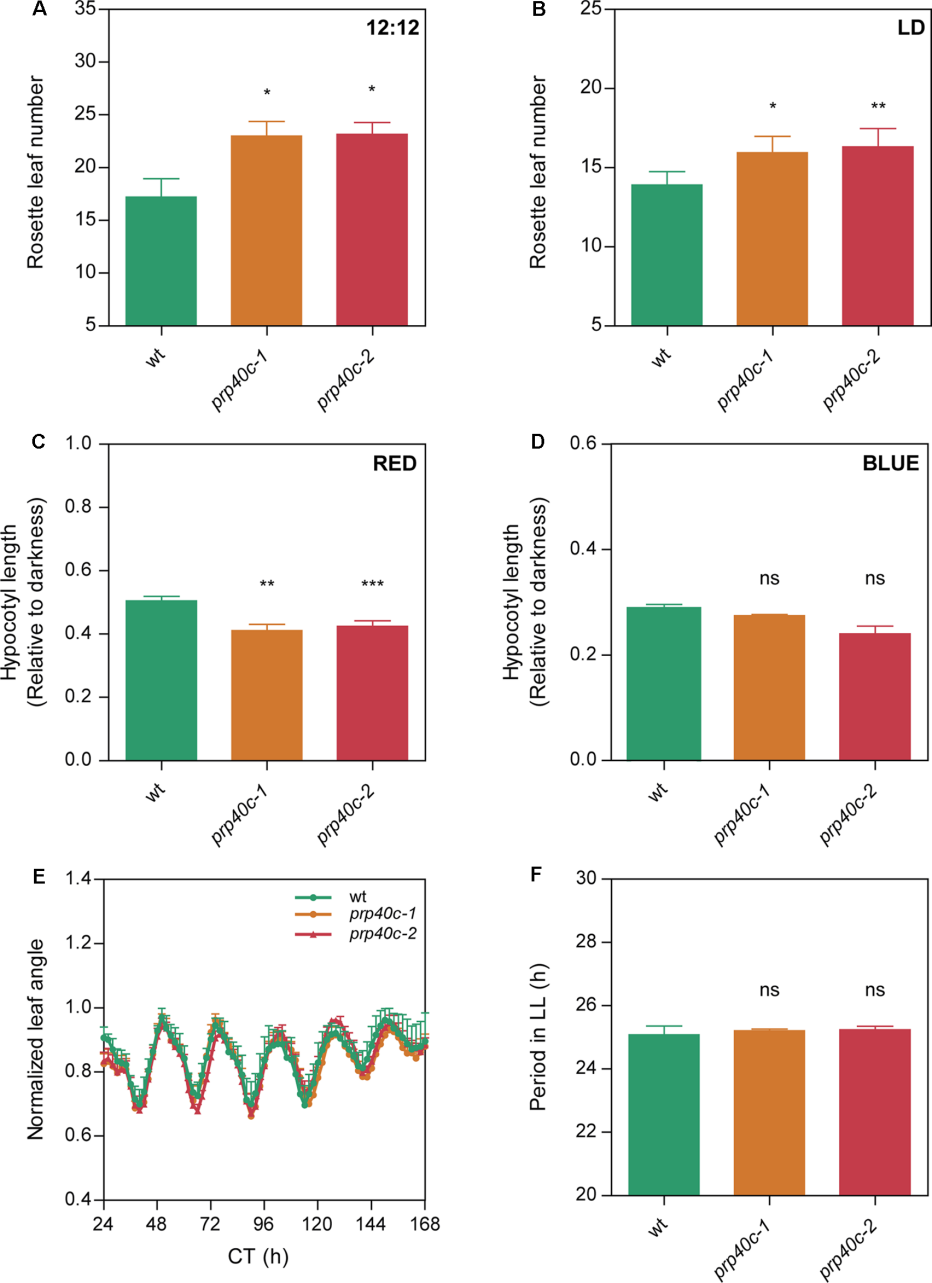
PRP40C plays a role in the photoperiodic control of flowering and photomorphogenesis. Flowering time measured as the number of rosette leaves at bolting in **(A)** 12-h light/12-h dark cycles (LD; 12:12), **(B)** long days (LD; 16:8). Hypocotyls of WT and PRP40C mutants grown under different wavelengths, measurements are expressed relative to the dark control. **(C)** Continuous red light. **(D)** Continuous blue light. **(E)** Circadian rhythms of leaf movement in continuous light (LL), after entrainment under long-day conditions. **(F)** Periods of circadian rhythms in leaf movement were estimated with BRASS 3.0 software. Error bars indicate SEM. Student’s *t*-test was performed between mutants and wild type (*significantly different, *p* ≤ 0.05; **significantly different, *p* ≤ 0.01; ***significantly different, *p* ≤ 0.001; ns, not significant).

In order to characterize the role of AtPRP40C (hereinafter referred to as PRP40C) using a reverse genetics approach, we identified plants with T-DNA insertions in the *PRP40C* loci, *prp40c-1* (SALK_148319), and *prp40c-2* (SALK_205357). Both mutants were genotyped to verify the homozygosity of the T-DNA insertion, and null expression of the wild-type mRNA was evaluated by RT-PCR in both mutant alleles. We then conducted an analysis of the effect of PRP40C in the control of several developmental and physiological processes. Initially, we did not observe severe phenotypic alterations in growth and morphology during the vegetative stage (**Supplementary Figure S1**). Then, we studied flowering time, a key developmental trait that marks the transition from the vegetative to the reproductive stage. We found that the *prp40c* mutants flower later than wild-type plants in both 12-h light/12-h dark and 16-h light/8-h dark long day photoperiods (**Figure 2A, B**). No delay in flowering time was observed in a short-day photoperiod or under continuous light (**Supplementary Figure S2**). Light perception and the circadian clock are two key factors that ensure proper flowering time regulation. To evaluate if any of these could be related to the late flowering phenotype, we analyzed photomorphogenic responses and clock function in the *prp40c* mutants. Seedling photomorphogenesis was assessed measuring the inhibition of hypocotyl elongation under different light treatments. Interestingly, *prp40c* mutants displayed shorter hypocotyls than wild-type plants under continuous red light, indicating hypersensitivity to this wavelength (**Figure 2C**). No differences were detected under continuous blue light (**Figure 2D**). We then assessed the effect of different photoperiods on hypocotyl elongation. No significant differences were detected between wild-type plants and *prp40c* mutants in any photoperiodic condition under white light (**Supplementary Figure S3**). Finally, we monitored circadian rhythms in leaf movements in wild-type plants as well as in *prp40c-1* and *prp40c-2* mutants. The rhythms observed for both mutant alleles were similar to those of wild-type plants, exhibiting a 24.5-h period (**Figure 2E, F**). These data suggest that the circadian function is unaffected, at least under standard growth conditions, in these plants. Collectively, our results indicate that PRP40C has a role in flowering time control, which could be associated, at least in part, with alterations in the red light signaling pathway.

### Impact of PRP40C on Genome-Wide Gene Expression and Pre-mRNA Splicing

**Figure 3:**
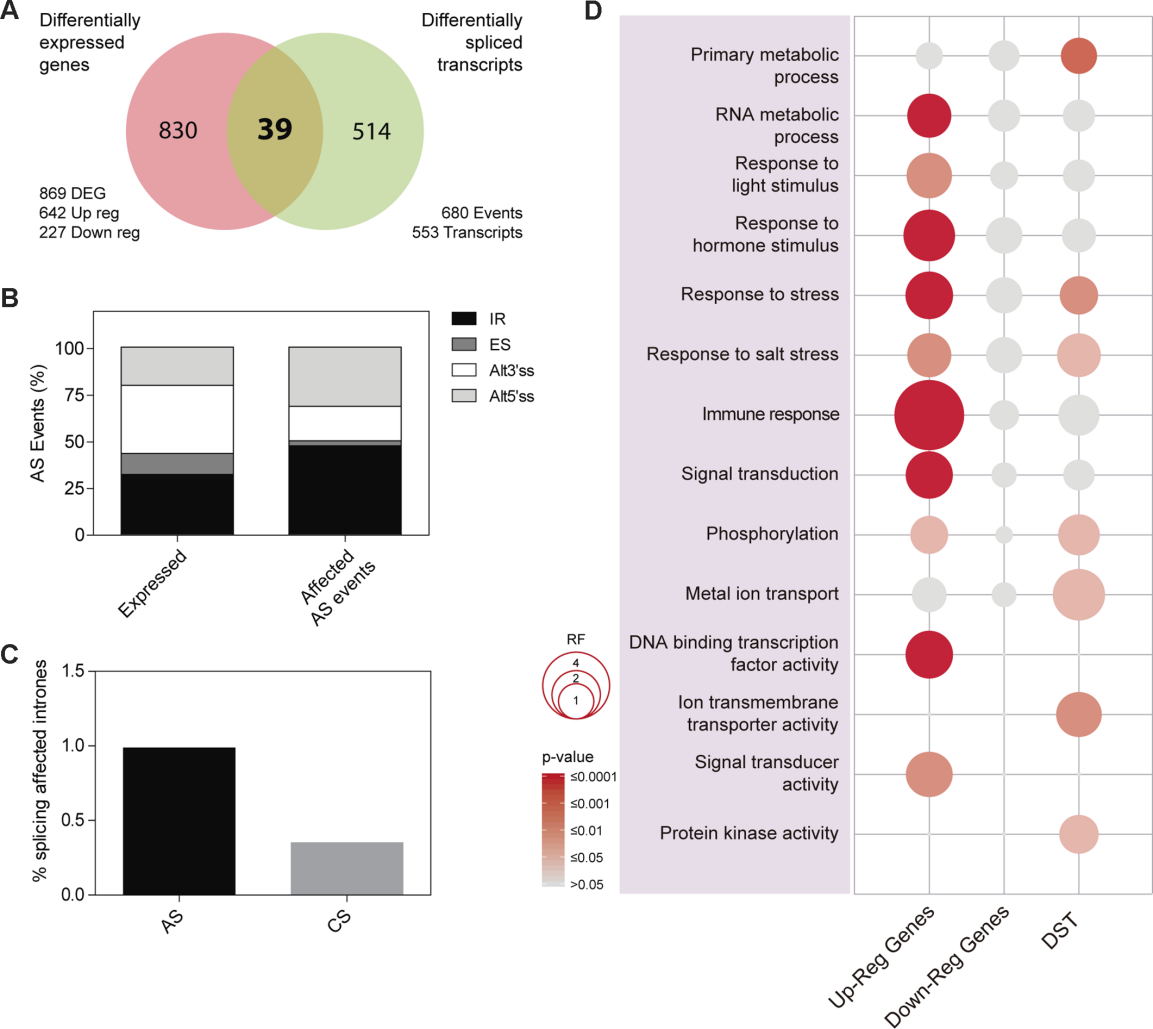
Impact of PRP40C on genome wide gene expression and pre-mRNA splicing. **(A)** Overlap between differentially expressed genes (DEG) and differentially spliced transcripts (DST). DEG are classified into up-regulated (Up-reg) and down-regulated (Down-reg) genes. **(B)** Relative frequencies of different alternative splicing (AS) types for all detected AS events; alt 3′ and alt 5′, alternative acceptor and donor splice sites, respectively; ES, exon skipping; IR, intron retention. **(C)** Percentage of introns showing increased retention in *prp40c-1* mutants in comparison with wild-type plants, introns were qualified as alternative (AS) or constitutive (CS). **(D)** Analysis of Gene Ontology (GO) enrichment comparing differentially expressed genes and differentially spliced transcripts. The color gradient represents adjusted *p* values and the differences in bubble size correlate with the enrichment factor.

In order to study the regulatory impact of PRP40C on gene expression, we analyzed the transcriptome of wild-type and *prp40c-1* mutant plants grown under standard nonstressful conditions (continuous white light at 22°C) using RNA-seq. We found 869 differentially expressed genes (DEG) in *prp40c-1* mutants relative to wild-type plants, 642 overexpressed (73.9%) and 227 underexpressed (26.1%). To characterize the role of PRP40C in pre-mRNA splicing, we evaluated both constitutive (CS) as well as alternative splicing (AS) events, which included annotated or novel AS events. We found a total of 680 differentially spliced transcripts (DST) in *prp40c-1* mutants when compared to wild-type plants. These events occurred on 553 transcripts, indicating that only a few transcripts had more than one differentially spliced event. Interestingly, only 39 transcripts were differentially expressed and differentially spliced at the same time (**Figure 3A**). We next studied the abundance of the different annotated AS events among the differentially spliced transcripts. In wild-type plants, the most abundant AS events were those associated with the use of alternative 3’ splice sites (Alt 3’SS, 36.4%), followed by intron retention (IR, 32%), alternative 5’ splice sites (Alt 5’SS, 20.3%), and exon skipping (ES, 11.2%). Among the annotated AS events affected in *prp40c-1*, we found an increase in the proportion of both IR events (IR, 47.4%) and alternative 5’ splice sites (Alt 5’SS, 31.6%) and a decrease in alternative 3’ splice sites (Alt 3’SS, 18.4%) and exon skipping events (ES, 2.6%), relative to their frequency in wild-type plants (**Figure 3B**). We also evaluated splicing of all introns present in expressed genes. Among the 105,555 introns analyzed, we detected alterations in splicing of 397 introns from which 18 were already annotated as alternatively spliced, and 379 had no previous evidence of being alternatively spliced (i.e., they were considered constitutively spliced introns). Interestingly, the proportion of increased intron retention events detected in the *prp40c-1* mutants among alternatively spliced introns was larger than the proportion observed among constitutively spliced introns (**Figure 3C**). This observation indicates that PRP40C acts as a splicing modulator rather than as an essential splicing factor. We also evaluated whether there was any change in the splice-site sequences of the intron retention events affected in *prp40c-1* mutants compared to the consensus sequence of all the introns belonging to genes expressed in our RNA-seq experiment. Interestingly, no significant deviation from the consensus sequence was observed for the donor splice site or for the acceptor splice site for the introns whose splicing was affected in *prp40c-1* mutants (**Supplementary Figure S4**). In order to get a broader landscape of the changes in the transcriptome induced by PRP40C, both the DEG and the DST were categorized into functional groups based on Gene Ontology (GO). Fourteen functional categories of our interest were examined in detail determining the representation factor for each category (data available in **Supplementary Table S2**). The representation factor (RF) is the number of overlapping genes observed in the selected group divided by the expected number of overlapping genes drawn randomly from two independent groups. Among the upregulated genes, we found a significant enrichment (representation factor > 1; *p* < 0.05) for categories corresponding to RNA metabolic process, response to light stimulus, response to hormone stimulus, response to stress, response to salt stress, immune response, signal transduction, phosphorylation, sequence-specific DNA binding transcription factor activity, and signal transducer activity. For downregulated genes, no enrichment was found among the categories studied. Among the differentially spliced genes, significant enrichment was found for categories such as primary metabolic process, response to stress, response to salt stress, phosphorylation, metal ion transport, ion transmembrane transporter activity, and protein kinase activity (**Figure 3D**). None of the categories studied here displayed a statistically significant under-representation (representation factor < 1; *p* < 0.05). These data taken together indicate that, instead of being a global transcription and pre-mRNA splicing regulator, PRP40C regulates transcription and splicing of a defined subset of transcripts particularly related to biotic and abiotic stress responses.

### PRP40C Plays a Role in Both Salt and Biotic Stress Tolerance

**Figure 4:**
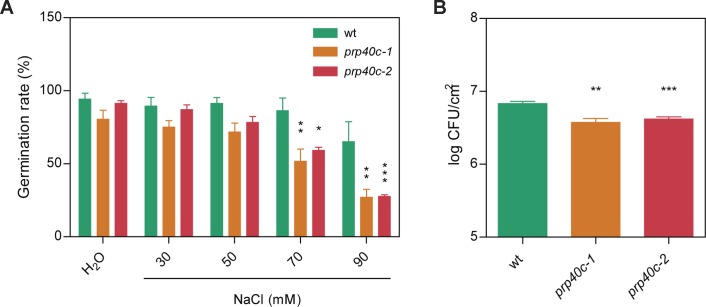
*prp40c* mutants show altered responses to both salt and biotic stress. **(A)** Seed germination rates in pure water and different NaCl concentrations were quantified. **(B)** Five- to six-week-old plants grown in short-day conditions (SD; 8-h light/16-h darkness) were infected by infiltration with *Pseudomona syringae* pv. *maculicolia* ES4326. Bacterial growth was assessed at 2 dpi. CFU, colony-forming units. Data represent the average of log-transformed bacterial growth. Error bars indicate SEM. An ANOVA followed by a Bonferroni comparison test was performed between mutants and wild type for the salt stress experiment; a Student’s *t*-test was performed between mutants and wild type for the infection assays (*significantly different, *p* ≤ 0.05; **significantly different, *p* ≤ 0.01; ***significantly different, *p* ≤ 0.001; ns, not significant).

**Figure 5:**
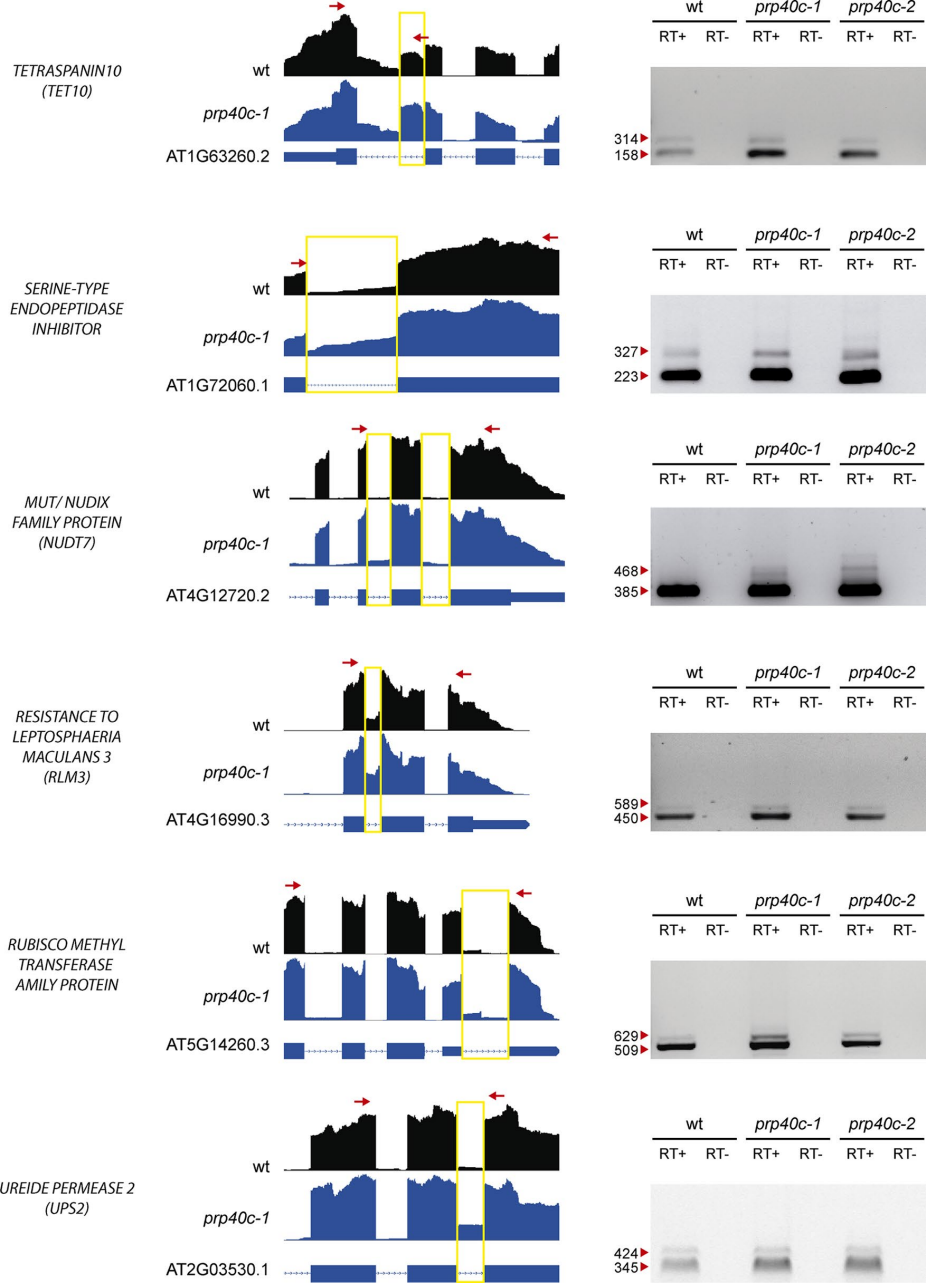
Analysis of alternative splicing events. RT-PCR validation of six events identified as alternatively spliced through RNA-seq. The read density map for each event evaluated is displayed (WT: black, *prp40c-1*: blue). A scheme describing each gene is displayed below the read density maps, with exons and introns displayed as boxes and lines, respectively. A yellow square encloses the measured event, while red arrows display the position of the oligos used for the RT-PCR measurement. An image of the agarose gel with the RT- PCR amplicons is displayed next to the read density maps. Red arrowheads indicate amplicon sizes. +, retrotranscriptase added; −, retrotranscriptase not added.

The global transcriptome analysis revealed a significant enrichment for genes related to salt and biotic stress responses among the differentially expressed and differentially spliced transcripts in *prp40c-1* mutants. We therefore performed physiological analyses of the mutant plants under both stress conditions. We first compared germination rates between wild-type plants and *prp40c* mutants under salt stress. There was no difference in germination rates between wild-type, *prp40c-1*, and *prp40c-2* seeds germinated on filter paper embedded with water, whereas the germination rate significantly decreased in *prp40c* mutants when the seeds were placed on a sodium chloride (NaCl) solution (**Figure 4A**). We next examined the root growth of wild-type plants and *prp40c* mutants under salt (NaCl), mannitol, and cold stress conditions, as well as survival rate under salt stress. No significant differences were found between genotypes in these experiments (**Supplementary Figure S5**). These results suggest that PRP40C is essential for proper germination under salt stress but is probably not involved in NaCl, mannitol, and cold tolerance in the early vegetative stage. In order to assess if *prp40c* mutants display alterations in response to biotic stress, we performed an infection assay with *Pseudomona syringae* pv. *maculicola* ES4326. Leaves from 5-week-old wild-type and *prp40c* plants were pressure infiltrated with the virulent bacterium. Bacterial growth assays at 2 days postinfection (2 dpi) revealed a significant degree of enhanced resistance in both *prp40c-1* and *prp40c-2* mutant alleles when compared to wild-type plants (**Figure 4B**). Finally, we confirmed by RT-PCR some of the alterations in splicing detected in transcripts related to abiotic and biotic stress in the RNA-seq experiment (**Figure 5**).

## Discussion

Proper control of gene expression and pre-mRNAs processing is crucial for eukaryotic organisms, and, indeed, many pre-mRNA processing events occur cotranscriptionally. PRP40 proteins appear to couple transcript elongation to the control of pre-mRNA splicing, but the global extent of their contribution to the regulation of gene expression networks, as well as their physiological roles, have only recently started to be characterized in higher eukaryotes (Munoz-Cobo et al., 2017). The founding member of this family (ScPRP40) was identified in yeast (Kao and Siliciano, 1996) and shown to be an essential splicing factor. Interestingly, PRP40 constitutes a small gene family in most eukaryotes, suggesting that duplication of the ancestral PRP40 gene followed by sequence divergence might have contributed to the evolution of novel molecular and/or physiological roles for some members of this protein family (Becerra et al., 2016). A comparison between PRP40s from *Saccharomyces cerevisiae*, *Homo sapiens*, and *A. thaliana* showed that A-type PRP40s are probably an ancestral feature of eukaryotic cells. Interestingly, AtPRP40C clusters with human TCERG1/CA150, but this clustering most likely reflects the divergent sequences in this group compared to other family members, rather than a common evolutionary origin. Still, C-type PRP40s proved to be conserved across all plant species, including streptophyte algae. This prompted us to explore a possible role for AtPRP40C as a modulator of gene expression programs, which could act linking transcription and splicing to optimize plant growth and development.

Loss-of-function *prp40c* mutants were viable and showed no severe phenotypical alterations compared to wild-type plants, suggesting that indeed PRP40C is a modulator of gene expression networks rather than an essential splicing factor. Whether other *PRP40* genes are essential in *Arabidopsis* remains to be determined. It is possible that different members of this gene family play both redundant as well as specific functions in the control of gene expression and that the essentiality of the *PRP40* gene family could only be observed in a triple mutant lacking all *PRP40* homologues.

In support of a modulatory function for PRP40C, *prp40c* mutants displayed a late flowering phenotype only under long-day photoperiods, revealing that the mutant does not have a global developmental alteration, but rather a defect in the environmental control of specific developmental traits. Furthermore, the finding that *prp40c *mutants only flower late under long-day conditions reveals that the photoperiodic flowering pathway may be specifically affected by this mutation; this well-known signaling pathway is controlled by light perception and the circadian clock through their effects on gene expression. *prp40c* mutants in fact exhibit hypersensitivity to red light but not alterations in circadian clock function, suggesting that a defect in light signaling rather than in time measurement could be associated with its altered flowering time phenotype.

To analyze the molecular cause of the late flowering phenotype, we looked for genes related to the control of the photoperiodic flowering pathway among the DEG in the mutant. We found four candidates: AT1G01060 (*LHY*), AT2G46830 (*CCA1*), AT1G25560 (*TEM1*), and AT1G68840 (*TEM2*). Given that the circadian function was not affected in the mutant, the flowering phenotype was most likely due, at least in part, to the overexpression of *TEM1* and *TEM2*, rather than to changes in *LHY* and *CCA1* clock genes (**Supplementary Figure S6**). In fact, the transcription factors TEM1 and TEM2 are floral repressors of the photoperiodic and the gibberellin flowering induction pathways (Osnato et al., 2012; Matias-Hernandez et al., 2014).

Almost three quarters of the differentially expressed genes in *prp40c-1* mutants were upregulated when compared to the wild type, suggesting that PRP40C may be acting as a negative transcriptional regulator. It has been proposed that HsTCERG1/CA150 is a promoter-specific negative regulator of RNA Pol II transcription elongation, and it is possible that plant PRP40C proteins have a similar repressive role (see **Supplementary Table S2**) (Sune et al., 1997; Sune and Garcia-Blanco, 1999).

Regarding alterations in pre-mRNA splicing in *prp40c-1* mutants, we detected 680 events as differentially spliced between mutant and wild-type plants. Although this is a significant number of differentially spliced events, it is relatively small compared with the numbers of altered events that we have previously observed in other *Arabidopsis* mutants affected in core spliceosomal components, such as *lsm5* (6,049 events) and *lsm4* (6,377 events), or in mutants affected in spliceosome assembly, such as *gemin2* (6680 events) (Perez-Santangelo et al., 2014; Schlaen et al., 2015). Among the different types of annotated AS events, IR was clearly overrepresented in the group of events that were differentially spliced between *prp40-c* mutants and wild-type plants. In addition, *prp40c* had a stronger impact on the splicing of alternative compared to constitutive introns. These results, taken together, suggest that AtPRP40C is a splicing modulator, which affects a specific subset of splicing events, rather than an essential splicing factor.

In contrast to what was reported for HsPRPF40B, we found no significant deviations in the sequences of the global consensus donor and acceptor splice sites compared to the sequences of donor or acceptors sites present in the differentially spliced events (i.e., the affected events were not associated with weak splice site signals). This observation suggests that AtPRP40C may contribute to the regulation of pre-mRNA splicing events through a mechanism that is at least partially different from its previously proposed role in the stabilization of weak RNA–RNA interactions (Becerra et al., 2015).

When DEG and the DST were categorized into functional groups several interesting results came to light. Among the DEG, upregulated genes were enriched in several GO categories (**Supplementary Table S2**). Some of the overrepresented categories may help to explain, at least in part, some of the phenotypes observed in the mutants, such as red light hypersensitivity (Response to light stimulus) and the splicing alterations (RNA metabolic process). This suggests that some of the splicing alterations observed in the *prp40c* mutants could take place through the differential expression of other splicing regulators rather than through a direct action of PRP40C on pre-mRNA processing.

Other GO terms significantly overrepresented were immune response (RF = 4.45), response to hormone stimulus (RF = 2.4), and response to stress (RF = 1.9). Particularly, we noticed that among the 34 upregulated genes related to the immune response ontology, 21 (60%) were also related to signal transduction (RF = 1.9). Among the DST signal-transduction related genes that were affected, categories such as phosphorylation (RF = 1.45) and protein kinase activity (RF = 1.38) were significantly enriched. Abiotic stress-related GO categories such as salt stress (RF = 1.73), metal ion transport (RF = 2.46), and ion transmembrane transporter activity (RF = 1.80) were also significantly enriched among DST. The ion-transport related categories were enriched among the DST but not among DEG, suggesting a level of specificity for AtPRP40C in splicing regulation.

Both biotic and abiotic stresses were differentially affected in *prp40c* mutants when compared to wild-type plants. Interestingly, seed germination was affected in the mutants under salt stress, but growth was not affected by this stress at the vegetative stage. In addition, cold stress during the early vegetative stage did not affect *prp40c* root growth. According to the data available in the Bio-Analytic Resource for Plant Biology of the University of Toronto (Toronto BAR—http://www.bar.utoronto.ca/), AtPRP40C expression is highest at the dry seed stage (**Supplementary Table S3**), which may explain why PRP40C plays a role in salt tolerance during germination. Nonetheless, a more thorough evaluation of stress tolerance phenotypes throughout a variety of life stages is needed to unveil in more detail the regulatory role of PRP40C in abiotic stress responses. On the other hand, *prp40c* mutants proved to be more resistant to *P. syringae* infection. We found that 15 out of the 34 DEG (44%) grouped in the immune response GO were TIR-NBS-LRR (Toll interleukin1 receptor–nucleotide binding site–leu-rich repeat) class of R (resistance) proteins, and some of these TIR-NBS-LRR proteins are known to enhance disease resistance when overexpressed (Parker et al., 1996; Tang et al., 1999; Stokes et al., 2002). These data suggest that AtPRP40C may regulate this specific group of genes negatively in order to orchestrate an appropriate defense response. Interestingly, according to the GENEVESTIGATOR database, the expression of the *PRP40C* gene appears to be modulated by both abiotic (salt and cold) as well as biotic (*P. syringae*) stresses, suggesting that PRP40C acts as a node within the stress response gene regulatory network in *Arabidopsis* (**Supplementary Figure S7**) (Hruz et al., 2008).

In summary, our work constitutes the first physiological and molecular characterization of a member of the PRP40 protein family in plants and the most thorough characterization of the global effects of PRP40 on gene expression and pre-mRNA splicing in eukaryotic organisms. We provide evidence that PRP40C is an important factor linking the regulation of gene expression programs to the modulation of plant growth, development, and biotic and abiotic stress responses. We propose that *PRP40C* evolved from an ancestral *PRP40* gene that had an essential role in the control of pre-mRNA splicing, into a global modulator of gene expression and splicing that targets specific genes and processes to improve physiological adjustments to environmental challenges. Although it is tempting to speculate that PRP40C modulates gene expression by coupling the regulation of transcription rates to the control of pre-mRNA splicing, its precise mechanism of action remains to be determined.

## Data Availability

The datasets generated for this study can be found in the Gene Expression Omnibus (https://www.ncbi.nlm.nih.gov/geo/query/acc.cgi?acc=GSE129932).

## Author Contributions

Most of the investigation and experiments were performed by CEH and MGH. Pathogen infection assay was performed by MJdL and PCR alternative splicing and qPCR differential expression assessment was performed by DC. RNA-Seq data analysis was performed by JI and sequence retrieval and phylogenetic analysisv was performed by SMG. Writing, review, and editing were done by CEH, SMG, and MJY. All authors read and approved the final manuscript.

## Funding

This research was funded by grants from Agencia Nacional de Promoción Científica y Tecnológica to MJY.

## Conflict of Interest Statement

The authors declare that the research was conducted in the absence of any commercial or financial relationships that could be construed as a potential conflict of interest.
