# Supplementary material for: A Role for Pre-mRNA-PROCESSING PROTEIN 40C in the Control of Growth, Development, and Stress Tolerance in Arabidopsis thaliana
Source: Front Plant Sci. 2019 Aug 13;10:1019. doi: 10.3389/fpls.2019.01019 (PMC6700278; doi:10.3389/fpls.2019.01019)
Supplement: Supplementary file 1 [file Image_1.pdf]

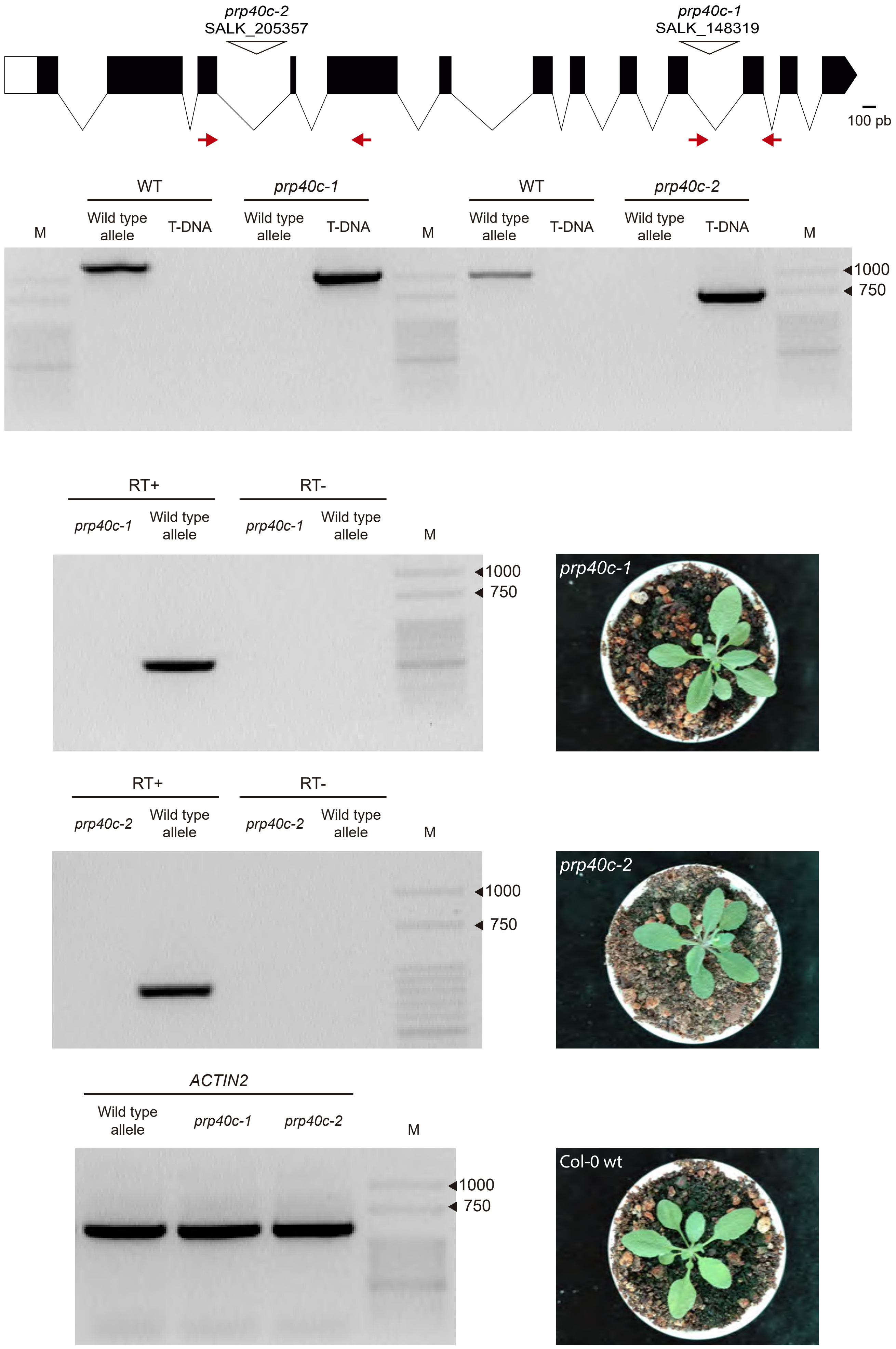

Supplementary Figure S1. *PRP40C* expression in different mutant backgrounds. Scheme of *PRP40C* showing the sites of T-DNAs insertions in the different mutant alleles. Exons and introns displayed as boxes and lines respectively, red arrows display the position of the oligo used for the genotyping. All mutant alleles have strongly reduced expression of the full-length mRNA, evaluated using primers flanking the T-DNA insertion. Plants were grown in MS in continuous light conditions for 12 days. Samples harvested were processed until cDNA synthesis. Transcript presence was determined by PCR. RT+: retrotranscriptase added, RT−: retrotranscriptase not added, M: molecular weight marker. Black arrows indicate amplicon sizes. Young rosettes of each genotype in the vegetative stage are displayed.
