# Supplementary material for: A Role for Pre-mRNA-PROCESSING PROTEIN 40C in the Control of Growth, Development, and Stress Tolerance in Arabidopsis thaliana
Source: Front Plant Sci. 2019 Aug 13;10:1019. doi: 10.3389/fpls.2019.01019 (PMC6700278; doi:10.3389/fpls.2019.01019)
Supplement: Supplementary file 2 [file Image_2.pdf]

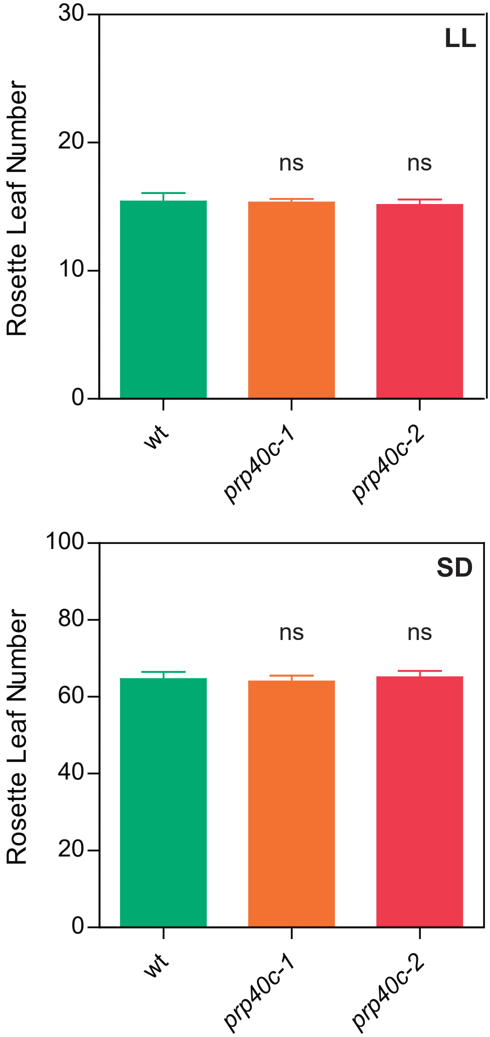

Supplementary Figure S 2. Characterization of PRP40C in the control of flowering. Flowering time measured as the number of rosette leaves at bolting in continuous white light (LL) and short days (SD; 8 h light/16 h darkness). Error bars indicate SEM. Student's t-Test was performed between mutants and wild-type (ns: not significant).
