# Supplementary material for: A Role for Pre-mRNA-PROCESSING PROTEIN 40C in the Control of Growth, Development, and Stress Tolerance in Arabidopsis thaliana
Source: Front Plant Sci. 2019 Aug 13;10:1019. doi: 10.3389/fpls.2019.01019 (PMC6700278; doi:10.3389/fpls.2019.01019)
Supplement: Supplementary file 3 [file Image_3.pdf]

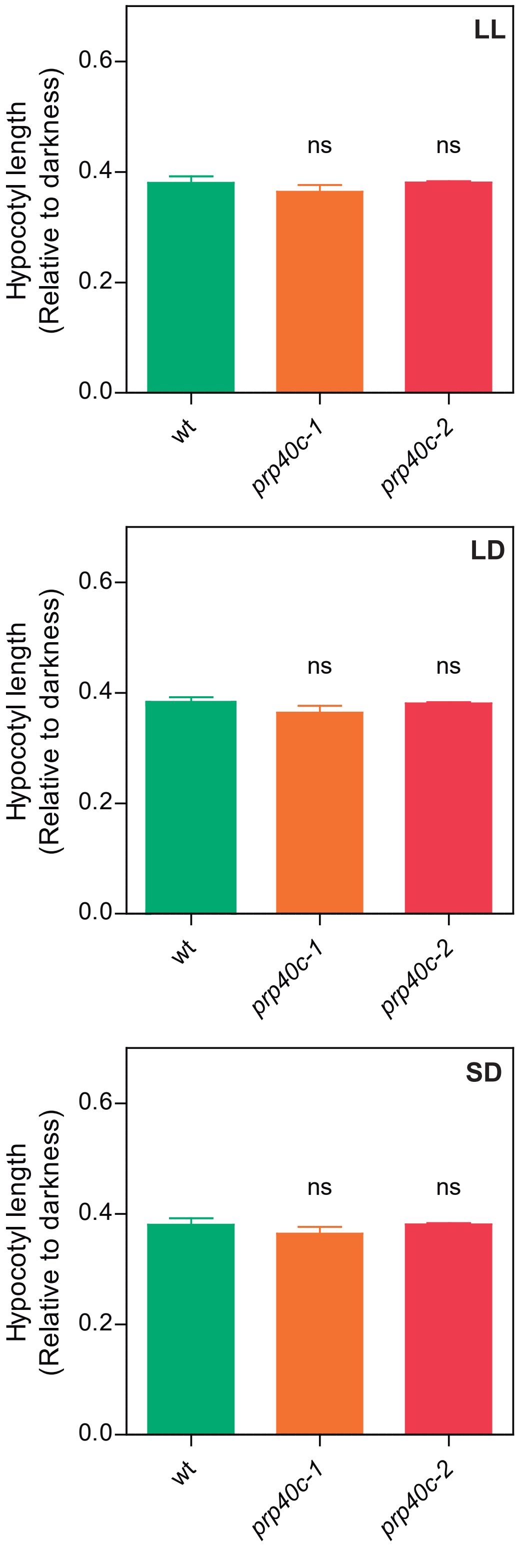

Supplementary Figure S3. Role of PRP40C in the control of photomorphogenesis. Hypocotyls of WT and *prp40c* mutants grown under different photoperiods; measurements are expressed relative to the dark control. Continuous white light (LL); Long-day photoperiod in white light (LD; 16 h light/8 h darkness); Short-day photoperiod in white light (SD; 8 h light/16 h darkness). Error bars indicate SEM. Student's t-Test was performed between mutants and wild-type (ns: not significant).
