# Supplementary material for: A Role for Pre-mRNA-PROCESSING PROTEIN 40C in the Control of Growth, Development, and Stress Tolerance in Arabidopsis thaliana
Source: Front Plant Sci. 2019 Aug 13;10:1019. doi: 10.3389/fpls.2019.01019 (PMC6700278; doi:10.3389/fpls.2019.01019)
Supplement: Supplementary file 4 [file Image_4.pdf]

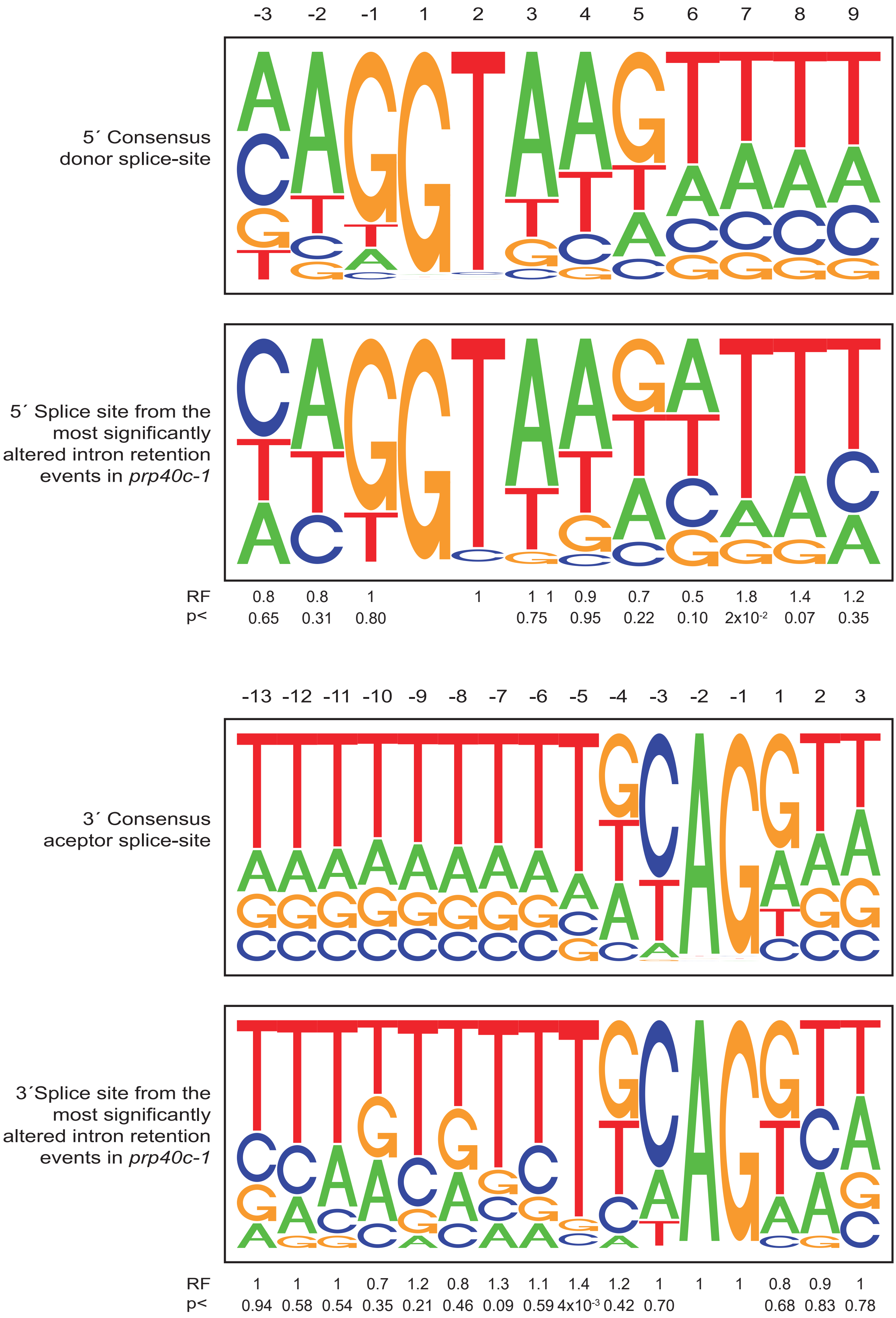


**Supplementary Figure S4.** Bioinformatic analysis of 5’ and 3’ donor splice-site sequences. Pictograms showing the frequency distribution of nucleotides at the 5′ splice site of 5’ donor splicing site of 30,142 Arabidopsis introns sequenced in our experiment. 5’ donor splicing site of the most significantly intron retention events whose splicing were altered in prp40c-1. 3’ donor splicing site of 30,142 Arabidopsis introns sequenced in our experiment. 3’ donor splicing site of the most significantly intron retention events whose splicing were altered in prp40c-1. The representation factor (RF) is the frequency in the data set of interest divided by the total frequency. For each RF a p-value was calculated using the hypergeometric test.
