# Supplementary material for: A Role for Pre-mRNA-PROCESSING PROTEIN 40C in the Control of Growth, Development, and Stress Tolerance in Arabidopsis thaliana
Source: Front Plant Sci. 2019 Aug 13;10:1019. doi: 10.3389/fpls.2019.01019 (PMC6700278; doi:10.3389/fpls.2019.01019)
Supplement: Supplementary file 5 [file Image_5.pdf]

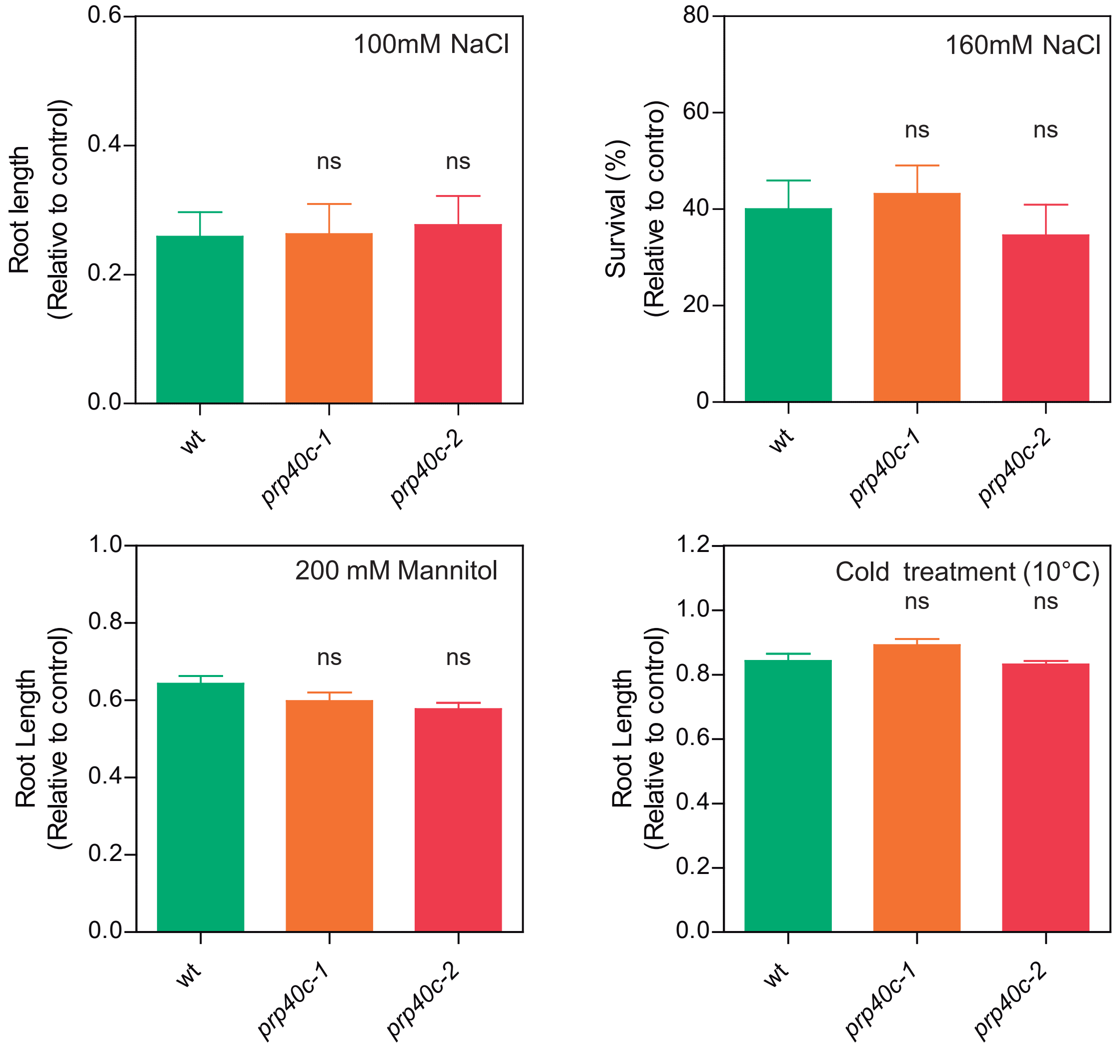

Supplementary Figure S5. *prp40c* mutants do not display susceptibility to salt stress at the vegetative growth stage. Salt tolerance was assessed through the analysis of survival rate of 14 days-old plants growth in regular MS medium or in MS containing 160mM NaCl. Comparison of root growth on MS medium with 100 mM NaCl, 200 mM Mannitol and cold treatment (10°C). Root growth was measured relative to control plants. Error bars indicate SEM. Student's t-Test was performed between mutants and wild-type (ns: not significant).
