# Supplementary material for: A Role for Pre-mRNA-PROCESSING PROTEIN 40C in the Control of Growth, Development, and Stress Tolerance in Arabidopsis thaliana
Source: Front Plant Sci. 2019 Aug 13;10:1019. doi: 10.3389/fpls.2019.01019 (PMC6700278; doi:10.3389/fpls.2019.01019)
Supplement: Supplementary file 6 [file Image_6.pdf]

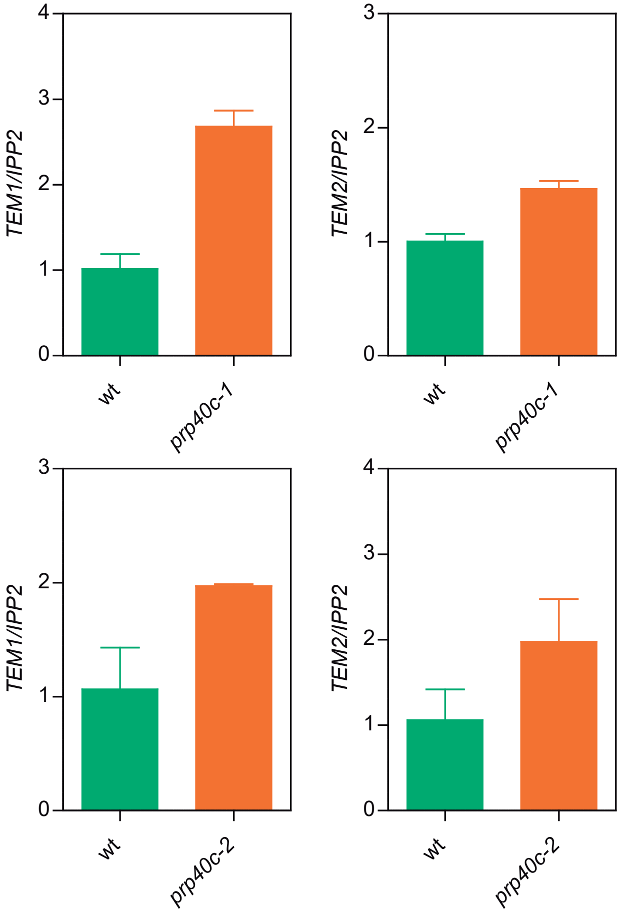

Supplementary Figure S6. RT-qPCR gene expres sion quantification of photoperiodic flowering pathway regulators. Expression levels were measured by qPCR for the photoperiodic flowering pathway regulators *TEM1* and *TEM2*. Three biological replicates were measured. Error bars indicate SEM. Student's t-Test was performed between mutants and wild-type (significantly different, *: $p \leq 0.05$).
