# Supplementary material for: A Role for Pre-mRNA-PROCESSING PROTEIN 40C in the Control of Growth, Development, and Stress Tolerance in Arabidopsis thaliana
Source: Front Plant Sci. 2019 Aug 13;10:1019. doi: 10.3389/fpls.2019.01019 (PMC6700278; doi:10.3389/fpls.2019.01019)
Supplement: Supplementary file 7 [file Image_7.pdf]

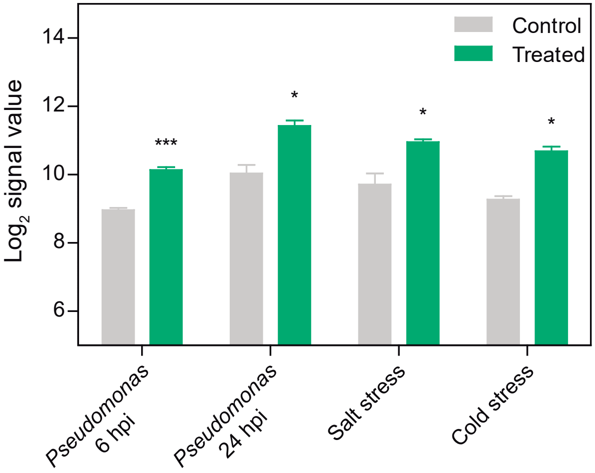

Supplementary Figure S7. *PRP40C* expression in response to biotic and abiotic stress. *PRP40C* expression in biotic and abiotic stress was analyzed using the GENEVESTIGATOR database. Expression of *PRP40C* 6 hours post infection (hpi) with Pseudomonas syringae pv. tomato DC3000 infected Col-0 plants and control plants was taken from the experiment: Response to virulent, avirulent, type III-secretion system deficient and nonhost bacteria (https://www.arabidopsis.org/servlets/TairObject?type=expression_set_full&id=1007966202) and expression of *PRP40C* 24 hours post infection (hpi) with Pseudomonas syringae pv. tomato DC3000 infected plants and control plants from the experiment: Genome-wide transcriptional analysis of the compatible A. thaliana-P. syringae pv. tomato DC3000 interaction (GSE5520). Expression of *PRP40C* in salt stress was taken from the expriment: A spatio-temporal understanding of growth regulation during the salt-stress response (GSE46205). Expression of *PRP40C* in cold stress from the experiment: Response to cold, plate grown plants (GSE5534). Error bars indicate SEM. Student's t-Test was performed between mutants and wild-type (*: significantly different, $p \leq 0.05$; ***: significantly different, $p \leq 0.001$).
